# BMI-Stratified Physiological Responses to a 12-Week Mat Pilates Intervention on Body Composition, Cardiometabolic Risk Factors, and Autonomic Function in Middle-Aged Women: An Exploratory Secondary Analysis

**DOI:** 10.3390/metabo16080517

**Published:** 2026-07-23

**Authors:** Won-Sang Jung, Won-Je Kim, Eunjoo Lee, Hana An, Hun-Young Park

**Affiliations:** 1 Department of Senior Exercise Prescription, Future Career College, Dongseo University, Busan 47011, Republic of Korea; jws1197@dongseo.ac.kr (W.-S.J.); hana1259@dongseo.ac.kr (H.A.); 2 Department of Sports Medicine and Science, Graduate School, Konkuk University, Seoul 05029, Republic of Korea; jasonkim0303@konkuk.ac.kr (W.-J.K.); eunjooo@konkuk.ac.kr (E.L.); 3 Physical Activity and Performance Institute (PAPI), Konkuk University, Seoul 05029, Republic of Korea

**Keywords:** mat Pilates, obesity, cardiometabolic risk, flow-mediated dilation, heart rate variability, middle-aged women

## Abstract

**Background/Objectives**: Mat Pilates is widely recommended for middle-aged women, but whether baseline obesity modifies its effects remains unclear. This study compared responses to a 12-week mat Pilates programme between non-obese and obese middle-aged women across body-composition, cardiovascular, blood-metabolic, haemorheological and autonomic endpoints. **Methods**: In an exploratory secondary analysis of a completed, uncontrolled single-arm intervention cohort, de-identified pre- and post-intervention data from 32 premenopausal women (47.5 ± 7.6 years) were stratified by BMI into non-obese (NOG; <25 kg/m^2^; *n* = 14) and obese (OG; ≥25 kg/m^2^; *n* = 18) groups using WHO Asia-Pacific criteria. All completed identical 60 min sessions three times weekly for 12 weeks, with intensity progressed every four weeks. Outcomes were analysed with mixed-design ANOVA and Bonferroni-corrected paired *t*-tests. **Results**: Group × time interactions were significant for body weight, fat mass and percent body fat (*p* < 0.05). Flow-mediated dilation rose in both groups (NOG +15.6%, OG +29.7%; *p* < 0.05). Group × time interactions were non-significant for blood pressure, lipids and haemorheology; obesity-dependent claims are therefore restricted to the outcomes with significant interactions (body weight, fat mass, percent body fat). For the remaining outcomes, within-group changes reached significance only in the OG (total cholesterol, LDL-cholesterol, aggregation index, critical shear stress) or only in the NOG (diastolic and mean arterial pressure) but did not differ significantly between groups. **Conclusions**: Following the programme, endothelial function improved in both groups, whereas body-composition responses differed significantly by obesity status. Because this uncontrolled secondary analysis lacked a non-exercising control group, these associations cannot be attributed causally to mat Pilates. The apparent BMI dependence was not reproduced when adiposity was modelled continuously (baseline BMI, percent body fat or fat mass), so all between-group contrasts are hypothesis-generating. Adequately powered randomised controlled trials with a non-exercising comparator and objective monitoring of diet, non-exercise activity, sleep and menstrual-cycle phase are needed before BMI-dependent adaptations to mat Pilates can be considered established.

## 1. Introduction

In middle-aged women, obesity tends to cluster with insulin resistance, dyslipidaemia, endothelial dysfunction, altered haemorheology and autonomic imbalance, and together these raise the long-term risk of type 2 diabetes and atherosclerotic cardiovascular disease [1,2,3]. The derangements share a common pathophysiological core. Chronic low-grade inflammation and oxidative stress reduce endothelial nitric oxide (NO) bioavailability, increase sympathetic outflow to the vasculature and adipose tissue, and impair erythrocyte deformability and plasma viscosity [4,5]. This profile is further shaped by the hormonal transition of perimenopause, which accelerates visceral fat accumulation and the loss of lean and bone mass [6,7]. Asian women warrant particular attention: at any given BMI they carry more visceral adipose tissue and develop cardiometabolic complications at lower body weight than European women—the rationale for the WHO Asia-Pacific obesity cut-off of BMI ≥ 25 kg/m^2^ rather than the conventional ≥30 kg/m^2^ [8,9]. Finding exercise that delivers real cardiometabolic gain in this group, and that is sustainable in community settings, remains a practical priority.

Pilates has become a popular low-impact option for women in midlife. It integrates core stabilisation, controlled breathing, postural alignment and submaximal resistance [10], and improves body composition, lipid profile, blood pressure and selected fitness measures in overweight and obese populations [11,12,13,14]. The case for vascular benefit rests on three mechanisms that are well described for higher-intensity exercise but are only beginning to be tested in Pilates. Rhythmic diaphragmatic breathing and intermittent isometric core contractions generate repeated low-magnitude oscillations in intrathoracic pressure and venous return, exposing the endothelium to patterns of laminar shear stress that have been reported to up-regulate endothelial nitric oxide synthase (eNOS) and raise NO bioavailability [15,16,17]; NO bioavailability, shear-rate stimulus, oxidative-stress and inflammatory markers, autonomic outflow and erythrocyte membrane properties were not measured in the present study, so all such pathways are advanced as possible explanations consistent with the observed pattern rather than as mechanistic evidence arising from these data. Resolving them would require direct measurement rather than inference, and recent work indicates that pathway-level blood metabolomics—summarising metabolites into domains such as lipid metabolism and inflammation/oxidative stress—can capture lifestyle-associated metabolic variation and relate it to functional performance more sensitively than a small panel of routine analytes [18]. Applying such a framework to Pilates training would be a logical next step. Sustained controlled breathing also modulates baroreflex sensitivity and lowers resting sympathetic outflow, which could in turn affect both blood pressure and heart rate variability. Finally, repeated low-intensity shear exposure has been shown to enhance erythrocyte NO synthase activity, erythrocyte deformability and microvascular perfusion [19,20], a plausible biological link between Pilates and haemorheological adaptation. Twelve weeks is the conventional minimum in this literature, since conduit-artery adaptation to repeated shear typically needs 8–12 weeks of training to appear [17]. Two recent contributions are especially relevant. A topical review by Bertoli and colleagues catalogued 14 Pilates trials involving 582 overweight or obese participants and reported consistent improvements in anthropometric, metabolic and blood-pressure outcomes [21]. A 2024 network meta-analysis of five exercise modalities in postmenopausal women found that endurance, interval, resistance and hybrid training each enhanced FMD [22]; Pilates, however, was not included, an indication of how thin the direct vascular evidence for it remains in this population.

Three gaps persist despite this growing literature. The response to Pilates is rarely contrasted between non-obese and obese women under a single standardised protocol. That contrast matters biologically, because obese individuals carry a distinct profile of sympathetic activation, insulin resistance, microvascular impairment and oxidative stress [23,24,25]. These features lower baseline endothelial function, creating greater absolute room for improvement, but they may also blunt the adaptive response through persistent inflammatory drive [5], so whether the net result favours larger, equivalent or smaller adaptation in obese women cannot be predicted in advance. A second gap is the narrow outcome set of most Pilates trials, which centre on body composition and physical fitness and do not simultaneously profile DXA composition, vascular function (FMD and arterial stiffness), blood-metabolic markers, haemorheological parameters and full time- and frequency-domain HRV [26,27]. Without such an integrated profile it is hard to tell whether Pilates produces a coherent cardiometabolic shift or only piecemeal change. Third, the most directly comparable Korean cohort, in which our group recently examined online versus face-to-face Pilates in obese middle-aged women [13], has not been set against non-obese women trained under the same supervised programme.

From the mechanisms above we set out three hypotheses. (i) FMD would improve in both groups, with the larger relative gain in obese women given their lower baseline endothelial function and greater room for shear-mediated NO adaptation. (ii) Resting blood pressure would fall more clearly in non-obese women, in whom lower sympathetic drive and intact endothelial reserve permit a hypotensive response to low-intensity training, whereas blood pressure in obese women would be sustained by structural and neurohumoral mechanisms unresponsive to a 12-week Pilates dose. (iii) Body-composition and metabolic responses would diverge, with obese women needing additional dietary or higher-intensity stimulus to change adiposity while improving most in the markers where their baseline impairment is greatest, namely lipid profile and haemorheology. Because BMI stratification was applied post hoc rather than being prespecified in the original trial, these hypotheses and the associated between-group comparisons are exploratory and hypothesis-generating.

## 2. Materials and Methods

### 2.1. Study Design

**Figure 1:**
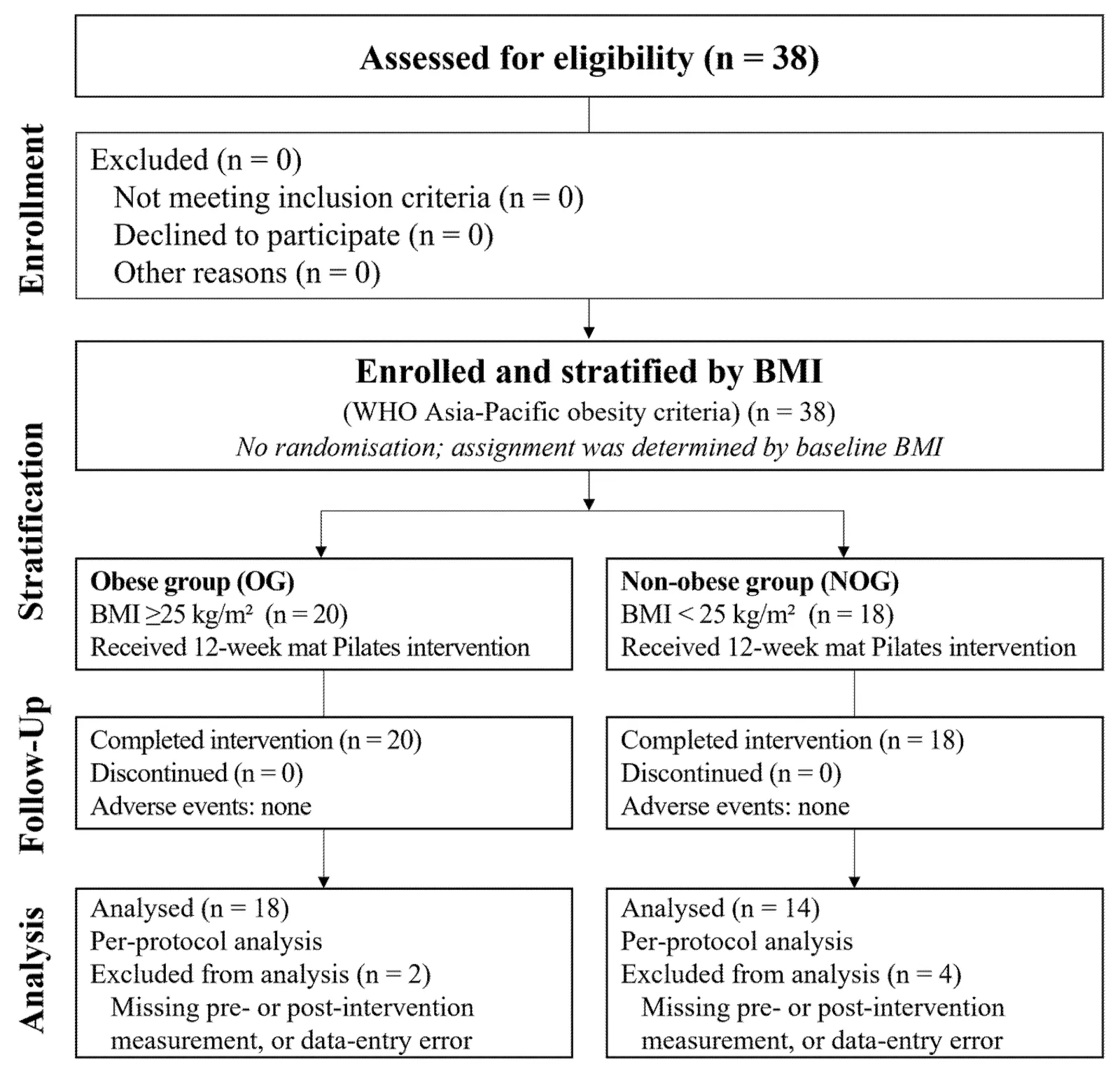
Participant flow through the secondary analysis. De-identified data from a completed 12-week prospective mat Pilates trial were restratified by baseline BMI (WHO Asia-Pacific criteria) for the present analysis. Six of the 38 original participants were excluded for missing pre- or post-intervention measurements or data-entry errors (four from the non-obese group and two from the obese group), leaving a final analytical sample of 32 (NOG, *n* = 14; OG, *n* = 18). BMI, body mass index; NOG, non-obese group; OG, obese group.

This is a secondary analysis of de-identified data from a completed 12-week prospective mat Pilates intervention trial. The original cohort was restratified by baseline obesity status to compare physiological responses between non-obese and obese middle-aged women trained under an identical protocol; obesity status was the between-subjects factor and time (pre versus post) the within-subjects factor. The question of interest was how non-obese and obese women, whose cardiometabolic profiles diverge in clinically meaningful ways and are known to shape exercise adaptation, respond to the same mat Pilates programme. BMI stratification was thus an analytical choice in the present study rather than a design feature of the original trial. This is therefore an exploratory secondary analysis of an uncontrolled, single-arm cohort: the original trial included no non-exercising control group, and obesity stratification was not planned a priori. Causal attribution of the observed changes to mat Pilates, and interpretation of between-group differences as causal effect modification by obesity, are consequently not warranted. The analysis was approved as a non-interventional study of de-identified data by the Institutional Review Board of Dongseo University, with a waiver of informed consent for secondary use. Participant flow is summarised in Figure 1.

### 2.2. Participants

De-identified pre- and post-intervention data were extracted from the original trial database, in which 38 premenopausal middle-aged women had completed a 12-week supervised mat Pilates programme. Participant recruitment and all pre- and post-intervention measurements for the original trial were carried out at Konkuk University in Seoul, Republic of Korea; the present secondary analysis was conducted at Dongseo University, Busan, which received only the de-identified dataset. The original cohort had been recruited in the Seoul metropolitan area through community announcements and posted notices. For the present analysis, participants were restratified by BMI using the WHO Asia-Pacific criteria [9] into a non-obese group (NOG; BMI < 25 kg/m^2^) and an obese group (OG; BMI ≥ 25 kg/m^2^). Six women were excluded for missing pre- or post-intervention measurements or data-entry errors (four from the non-obese group and two from the obese group), leaving a final analytical sample of 32 (NOG, *n* = 14; OG, *n* = 18). Because the original trial was single-arm and the BMI strata were formed post hoc, the diagram labels these steps as “stratified by baseline BMI” rather than “allocated”; no randomisation or allocation took place. Because the WHO Asia-Pacific cut-off of 25 kg/m^2^ groups normal-weight and overweight women within a single non-obese stratum, the NOG (BMI 22.9 ± 1.1 kg/m^2^) comprised upper-normal-weight and overweight participants, whereas the OG (BMI 28.0 ± 3.3 kg/m^2^) spanned a wider range of adiposity; this heterogeneity is acknowledged as a limitation of the dichotomised classification.

The original inclusion criteria were age 30–59 years; premenopausal status confirmed by self-reported regular menstruation; non-smoking; no current medication affecting body composition, lipid metabolism or autonomic function; and no regular structured exercise (defined as ≥150 min/week of moderate-intensity activity) in the preceding six months. Women were excluded if they were pregnant or lactating, postmenopausal, had had surgery within the previous six months, or had any musculoskeletal, cardiovascular, pulmonary or metabolic disease contraindicating moderate-intensity exercise. All participants had given written informed consent in the original trial after a full explanation of the procedures and possible risks. The original protocol was approved by the Institutional Review Board of Konkuk University, Seoul (approval no. 7001355-202112-HR-496) and registered with CRIS (KCT0008469); the present secondary analysis of de-identified data was reviewed and approved as a non-interventional study, with a waiver of informed consent for secondary use, by the Institutional Review Board of Dongseo University. Both studies complied with the Declaration of Helsinki. Baseline characteristics are given in Table 1.

Because this is a secondary analysis of an existing dataset, no a priori sample size calculation was performed for the BMI-stratified comparison. Rather than an achieved (post hoc) power value, which adds little beyond the reported effect sizes and confidence intervals, a sensitivity analysis was used to indicate the smallest effect the design could detect (G*Power 3.1; Heinrich Heine University, Düsseldorf, Germany). With the achieved sample (*n* = 32), α = 0.05 and 80% power, the 2 × 2 mixed-design ANOVA was sensitive to group × time interactions of f ≈ 0.25 (ηp^2^ ≈ 0.06) or larger. The large body-composition interactions observed here (ηp^2^ = 0.177–0.214) exceeded this threshold, whereas the smaller interaction effects on HRV and FMD (ηp^2^ ≈ 0.03) fell below it and are therefore regarded as underpowered; on these estimates, approximately 60 participants per group would be required to detect a group × time FMD interaction of ηp^2^ ≈ 0.03 at conventional power (this single figure is used consistently in the Discussion and Limitations).

### 2.3. Study Procedures

All testing was carried out at the Exercise Physiology Laboratory of Dongseo University. Pre-intervention measurements were obtained in the week before the programme began and post-intervention measurements in the week immediately after it ended. Sessions were scheduled between 07:00 and 11:00 to limit circadian variation; laboratory temperature was held at 22–24 °C and relative humidity at 40–60%. Data collection, offline image analysis and statistical analysis were performed by trained, certified evaluators. Blinding was partial: because obesity status is physically apparent, personnel could not be blinded to group allocation, whereas the assessor who performed offline FMD image analysis and the statistician were blinded to both group and time point. This partial blinding is considered when interpreting the results.

Participants avoided caffeine, alcohol and strenuous activity for 24 h before each visit and fasted for at least 8 h. Fasting fingertip blood was drawn first (between 07:00 and 09:00) for metabolic and haemorheological analyses. After a 30 min stabilisation period, resting blood pressure, arterial stiffness, brachial-artery endothelial function, body composition and HRV were measured in that fixed order. Aerobic capacity was assessed last, after a standardised snack and at least 60 min of recovery, to avoid carry-over effects.

### 2.4. Mat Pilates Intervention

Both groups completed the same 36-session mat Pilates programme: three sessions per week (Monday, Wednesday, Friday) for 12 weeks at a single training facility. Each 60 min session comprised a 10 min warm-up, 40 min of mat Pilates work guided by the six classical principles (breathing, centring, concentration, control, precision, flow) [28], and a 10 min cool-down. One certified instructor with more than 10 years of teaching experience supervised every session, keeping technique and pacing consistent across participants. Class size was capped at 6–8 so the instructor could correct movement quality individually.

**Figure 2:**
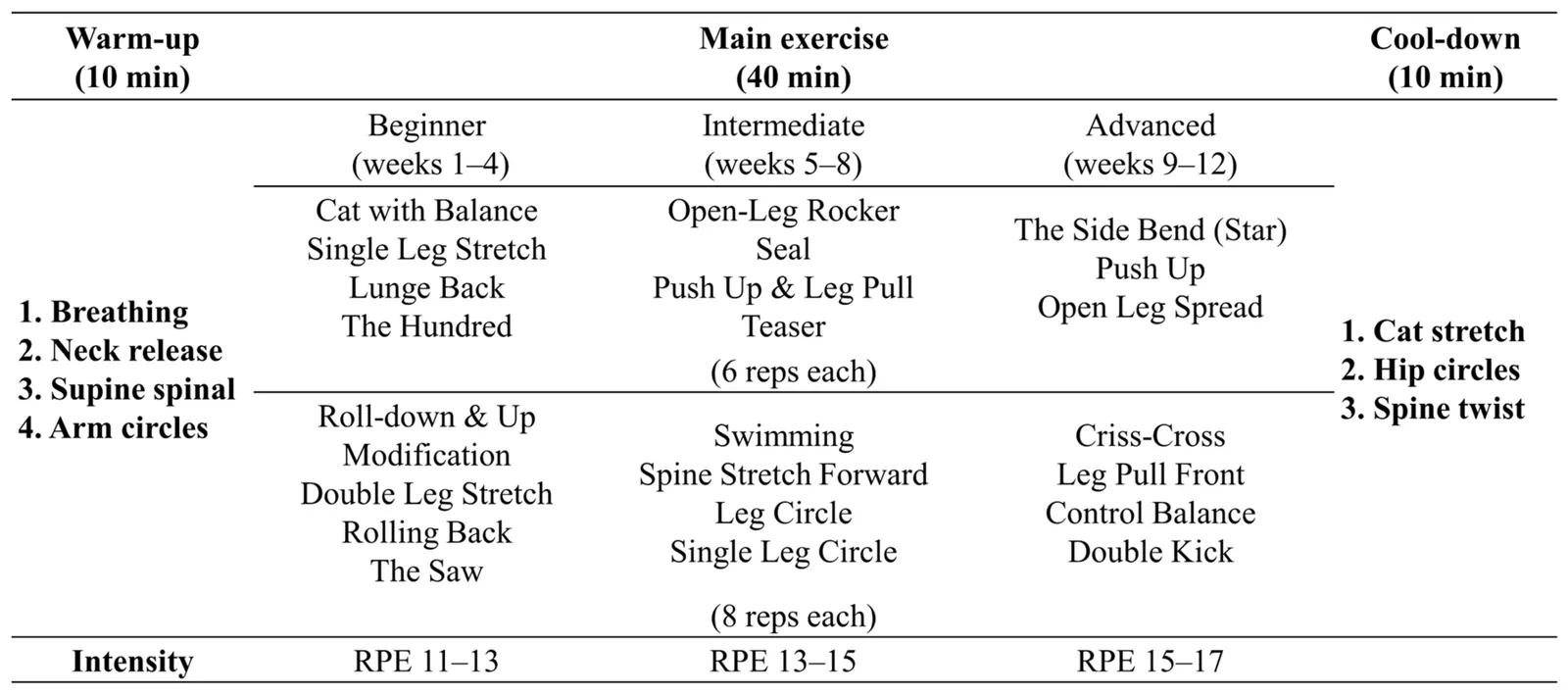
Twelve-week mat Pilates intervention programme. Each 60 min session comprised a 10 min warm-up, 40 min of main mat Pilates exercise and a 10 min cool-down, performed three times weekly for 12 weeks. Intensity was progressed every four weeks through increases in repetitions, range of motion and movement complexity. All sessions were supervised by the same certified Pilates instructor. RPE, rating of perceived exertion on the Borg 20-point scale.

Intensity was progressed every four weeks on the Borg 20-point RPE scale [29]: RPE 11–13 in the beginner stage (weeks 1–4), 13–15 in the intermediate stage (weeks 5–8) and 15–17 in the advanced stage (weeks 9–12). Exercise complexity, range of motion, repetitions (6 to 8 per movement) and movement tempo increased across stages while session duration stayed constant. The exercises used at each stage are listed in Figure 2. The instructor checked perceived exertion against the target range at the end of every session and adjusted load individually when participants reported values outside it.

Adherence was monitored in three ways. Attendance was recorded at every session, with a threshold of ≥85% (≥31 of 36 sessions) for inclusion in the per-protocol analysis. Participants also kept a daily Pilates diary noting session RPE, perceived enjoyment and any musculoskeletal discomfort. Face-to-face interviews at the end of each four-week stage identified barriers to adherence and reinforced motivation. Participants were asked to keep to their habitual diet and to avoid additional structured exercise, confirmed verbally at each monthly interview. No Pilates-related adverse events were reported. Adverse events were defined as any musculoskeletal injury, cardiovascular symptom or other health complaint arising during or within 24 h of a session, and were actively solicited at every session through the Pilates diary and at each monthly interview.

### 2.5. Outcome Measures

#### 2.5.1. Anthropometry and Body Composition

Standing height was measured to the nearest 0.1 cm with a wall-mounted stadiometer (YM-1; KDS, Seoul, Republic of Korea). DXA body composition, comprising total body mass (kg), fat-free mass (FFM, kg), fat mass (kg), percent body fat (%), bone mineral density (BMD, g/cm^2^) and bone mineral content (BMC, g), was obtained with a Primus DXA scanner (Osteosys, Seoul, Republic of Korea). Each whole-body scan was performed supine by a trained technician following the manufacturer’s standard protocol, with daily quality-control phantom measurements. The manufacturer-reported in vivo precision (coefficient of variation) for fat mass and percent body fat was applied when interpreting small pre–post differences, and changes smaller than the least significant change were not over-interpreted (reproducibility < 1.0% CV, giving a least significant change of approximately 2.8% by the 2.77 × CV criterion). Scans were standardised for an overnight fast, voided bladder, euhydration and time of day; menstrual-cycle phase was not standardised (see Section 4.7). BMI was calculated as body mass divided by height squared (kg/m^2^).

#### 2.5.2. Aerobic Exercise Capacity

Maximal oxygen uptake (VO_2_max, mL/kg/min) was estimated from a submaximal ramp protocol on a cycle ergometer (Aerobike 75XLIII; Konami, Tokyo, Japan) [13]. After participant-specific calibration for age, sex, weight and resting heart rate, pedalling began at 50 rpm and workload rose by 15 W/min until the participant reached 75% of age-predicted maximal heart rate (female HRmax = 205 − 0.75 × age). VO_2_max was estimated from the manufacturer’s regression equation (oxygen uptake = 9.386 × W + 289.6). Physical work capacity at 75% HRmax (PWC75%, W) was also recorded. Because this value is derived from a submaximal test, it is referred to as estimated VO_2_max throughout the manuscript.

#### 2.5.3. Resting Blood Pressure

Systolic and diastolic blood pressure (SBP, DBP) were measured at the right brachial artery with a validated automatic sphygmomanometer (ACCUNIQ BP210; SELVAS Healthcare, Daejeon, Republic of Korea) after at least 5 min of seated rest. Two consecutive measurements 1 min apart were averaged. Mean arterial pressure (MAP) was calculated as (SBP − DBP)/3 + DBP and pulse pressure (PP) as SBP − DBP.

#### 2.5.4. Arterial Stiffness

Brachial–ankle pulse wave velocity (baPWV, cm/s) and the ankle–brachial index (ABI) were measured bilaterally with a non-invasive vascular screening system (VP-1000 Plus; Omron Colin, Tokyo, Japan) in the supine position after a 5 min rest. Both right and left values were recorded.

#### 2.5.5. Endothelial Function

Flow-mediated dilation (FMD, %) of the right brachial artery was measured with a dedicated semi-automated ultrasound flow-mediated dilation system (UNEXEF38G; Unex, Tokyo, Japan) equipped with a high-frequency linear-array probe and continuous automated arterial wall-tracking, following published consensus guidelines [30]. Baseline brachial-artery diameter (mm) was recorded after 10 min of supine rest. A pneumatic forearm cuff was inflated to 50 mmHg above SBP for 5 min to induce reactive hyperaemia, and peak post-deflation diameter was recorded 60–90 s after cuff release. FMD was calculated as [(reactive hyperaemia diameter − baseline diameter)/baseline diameter] × 100. Brachial-artery diameter was recorded continuously from B-mode images and analysed with the integrated automated edge-tracking software of the UNEXEF38G system, and the peak diameter was identified from the continuous diameter trace; the 60–90 s window is reported because it captured peak dilation in this cohort, consistent with the cited consensus [30]. The probe was stabilised throughout acquisition, all images were analysed by a single assessor blinded to group and time point, and intra-rater reliability was not separately quantified in this secondary analysis and is acknowledged as a limitation; for guideline-adherent brachial FMD of this type, intra-observer reproducibility is typically an ICC ≥ 0.8 with a coefficient of variation of approximately 5–10%. The shear-rate stimulus was not quantified, which is acknowledged as a limitation.

#### 2.5.6. Blood-Metabolic Parameters

After an overnight fast (≥8 h), fingertip capillary blood was sampled for total cholesterol (TC, mg/dL), triglycerides (TG, mg/dL), high-density lipoprotein cholesterol (HDL-C, mg/dL), low-density lipoprotein cholesterol (LDL-C, mg/dL), free fatty acids (FFA, μEq/L), glucose (mg/dL) and insulin (μU/mL) using a Lipidocare analyser (SD BIOSENSOR, Suwon, Republic of Korea). Insulin resistance was indexed by the homeostasis model assessment (HOMA-IR = [glucose × insulin]/405). Manufacturer-reported analytical ranges and calibration procedures were applied for each analyte, the LipidoCare analyser (SD BIOSENSOR, Korea) is CRMLN-certified, with a manufacturer-reported analytical imprecision of <5% CV across analytes. Point-of-care capillary measurement of insulin, and HOMA-IR derived from it, have wider limits of agreement than venous laboratory assays; the insulin and HOMA-IR data are therefore interpreted only as group-level indicators rather than as diagnostic values. Free fatty acids were measured but showed below-threshold reliability on the capillary platform and were excluded from analysis, which is why FFA does not appear in the Results.

#### 2.5.7. Haemorheological Parameters

Whole-blood rheological properties were assessed with a microchip-based haemorheometer. (Rheoscan-D; Rheo Meditech Inc., Seoul, Republic of Korea). Blood was anticoagulated with K3-EDTA, and erythrocyte deformability (elongation index, EI) and aggregation (aggregation index, AI) were measured at 25 °C and 3 Pa shear stress within 4–6 h of collection. For EI, the sample was diluted in 5.5% polyvinylpyrrolidone (360 kDa) in phosphate-buffered saline (pH 7.4; 300 mOsmol/kg) and analysed with a D-test kit; AI was measured on 8 µL of whole blood with an A-test kit, following the manufacturer’s instructions. Critical shear-stress values are expressed in millipascals (mPa), the unit returned by the Rheoscan-D; the recorded magnitudes (approximately 340–520 mPa, i.e., 0.34–0.52 Pa) are consistent with reported erythrocyte disaggregation thresholds. The aggregation index (AI, %), the elongation index (EI) at a shear stress of 3 Pa as a marker of erythrocyte deformability, and the critical shear stress for disaggregation (mPa) were recorded.

#### 2.5.8. Heart Rate Variability

HRV was recorded with a Polar V800 chest-strap heart rate monitor (Polar Electro Oy, Kempele, Finland) sampling RR intervals at 1000 Hz, after 10 min of quiet supine rest, with continuous acquisition for 5 min during spontaneous breathing. Time-domain indices were the mean RR interval (ms), the standard deviation of NN intervals (SDNN, ms) and the root mean square of successive RR differences (RMSSD, ms). Frequency-domain indices were derived by fast Fourier transform of the resampled tachogram and comprised low-frequency power (LF, 0.04–0.15 Hz, normalised units), high-frequency power (HF, 0.15–0.40 Hz, normalised units) and the LF/HF ratio [31]. RR series were processed with Kubios HRV Standard software version 4.1 (Kubios Oy, Kuopio, Finland): ectopic beats and artefacts were identified and corrected by interpolation, the tachogram was resampled at 4 Hz and linearly detrended, and spectral power was obtained by fast Fourier transform using a Hann window, with recordings containing more than 5% corrected beats excluded. Respiratory rate was neither measured nor paced, a limitation given the strong respiratory influence on HF power. LF and HF are reported in normalised units (nu, where LF + HF = 100). Because the physiological specificity of the LF/HF ratio is debated, it is interpreted here as a coarse index of autonomic balance rather than as a direct measure of sympathovagal tone.

### 2.6. Statistical Analysis

Data are presented as mean ± standard deviation (SD). Normality was checked with the Shapiro–Wilk test and homogeneity of variance with Levene’s test before inferential analyses. Baseline characteristics were compared between groups with independent *t*-tests, or Mann–Whitney U tests where normality was violated. Intervention effects were evaluated with a two-way mixed-design ANOVA (group [NOG vs. OG] × time [pre vs. post]). Partial eta-squared (ηp^2^) was reported as the effect size, with values of 0.01, 0.06 and 0.14 taken as small, medium and large effects. When a significant group × time interaction or main effect emerged, Bonferroni-corrected paired *t*-tests identified within-group changes. Significance was set at *p* < 0.05 (two-tailed). Analyses were run in SPSS Statistics 25.0 (IBM Corp., Armonk, NY, USA). Because BMI stratification was a post hoc analytical decision, no confirmatory outcome hierarchy was prespecified and all analyses are exploratory. For descriptive purposes, body composition, FMD and resting blood pressure were treated as outcomes of primary interest and metabolic, haemorheological, arterial-stiffness and HRV variables as secondary/exploratory. The group × time interaction was the primary basis for inferring differential (BMI-dependent) responses; within-group paired tests were interpreted only as follow-up to a significant interaction or time main effect. Bonferroni correction was applied within each outcome family, and given the large number of correlated exploratory endpoints the results are treated as hypothesis-generating rather than confirmatory. In addition to *p*-values and partial eta-squared, 95% confidence intervals for the between-group difference in pre–post change are reported for the principal outcomes. Because the dichotomous WHO Asia-Pacific cut-off is a relatively crude index of adiposity and may obscure within-stratum heterogeneity, three sensitivity analyses were run in which the categorical grouping was replaced by continuous adiposity moderators: baseline BMI, baseline percent body fat and baseline fat mass. Each pre-to-post change score was regressed on the continuous moderator with adjustment for the corresponding baseline value, and the moderator coefficient (with 95% CI) was used to test whether the magnitude of change scaled with adiposity. A baseline-adjusted ANCOVA on follow-up values was additionally used to address baseline imbalance.

## 3. Results

### 3.1. Participant Characteristics

All 32 women in the analysis had completed the original 12-week intervention with mean attendance of 94.2% (range 86.1–100%) and were retained in the per-protocol analysis (Figure 1). Baseline characteristics are shown in Table 1. The groups did not differ in age (*p* = 0.464) or height (*p* = 0.721). (NOG 46.4 ± 8.0 years, OG 48.4 ± 7.4 years; all participants were 30–59-year premenopausal women and are described as middle-aged). As intended by the stratification, the OG had higher body weight, BMI and percent body fat than the NOG (all *p* < 0.001).

### 3.2. Body Composition and Aerobic Capacity

Body-composition and aerobic-capacity outcomes are presented in Table 2. Group × time interactions were significant for body weight (ηp^2^ = 0.129, *p* = 0.044), fat mass (ηp^2^ = 0.214, *p* = 0.008) and percent body fat (ηp^2^ = 0.177, *p* = 0.017). Post hoc tests showed that the NOG lost body weight (−1.5%, *p* < 0.05), whereas the OG gained fat mass (+2.2%, *p* < 0.05) and percent body fat (+1.6%, *p* < 0.05). BMI, FFM, BMD and BMC were unchanged in both groups. Neither VO_2_max nor PWC75% changed significantly, although the NOG showed a non-significant +10.4% trend in VO_2_max (*p* = 0.108).

### 3.3. Cardiovascular Function

Cardiovascular outcomes are summarised in Table 3. A main effect of time was significant for DBP (ηp^2^ = 0.123, *p* = 0.046) and FMD (ηp^2^ = 0.458, *p* < 0.001). DBP fell by 5.6% (*p* < 0.05) and MAP by 4.7% (*p* < 0.05) in the NOG, with no significant change in any blood-pressure variable in the OG. FMD rose in both groups, from 9.8 ± 2.6% to 11.3 ± 3.6% in the NOG (+15.6%, *p* < 0.05) and from 7.6 ± 2.5% to 9.8 ± 2.3% in the OG (+29.7%, *p* < 0.001). Baseline FMD was lower in the OG than in the NOG (*p* = 0.021); the relative improvement was larger in the OG, though the group × time interaction did not reach significance (*p* = 0.352). Baseline baPWV was higher bilaterally in the OG (*p* < 0.05), and neither baPWV nor ABI changed with the intervention in either group.

### 3.4. Blood-Metabolic Parameters

Blood-metabolic data are presented in Table 4. Main effects of time were significant for TC (ηp^2^ = 0.132, *p* = 0.038) and LDL-C (ηp^2^ = 0.121, *p* = 0.047). In the OG, TC fell by 6.0% (*p* < 0.05) and LDL-C by 9.0% (*p* < 0.05); the NOG showed no significant change. Baseline insulin (*p* = 0.023) and HOMA-IR (*p* = 0.026) were higher in the OG. Glucose, insulin and HOMA-IR were unchanged in both groups. None of the lipid or glucose-metabolism outcomes showed a significant group × time interaction, so the within-group pattern (significant change in the OG only) does not establish a statistically significant between-group difference.

### 3.5. Haemorheological Parameters

Haemorheological results are given in Table 5. Main effects of time were significant for AI (ηp^2^ = 0.135, *p* = 0.036) and critical shear stress (ηp^2^ = 0.376, *p* < 0.001). In the OG, AI decreased by 7.7% (*p* < 0.01) and critical shear stress by 25.5% (*p* < 0.001). The NOG showed a non-significant trend toward lower critical shear stress (−21.0%). The group × time interaction for AI approached significance (*p* = 0.056). EI was unchanged in both groups. The group × time interactions for aggregation index (*p* = 0.056) and critical shear stress (*p* = 0.429) were not significant, so these within-group improvements should not be read as significant between-group differences.

### 3.6. Heart Rate Variability

HRV data are presented in Table 6. No group × time interaction or main effect of time was significant for any time- or frequency-domain index. The NOG showed numerical trends toward higher SDNN (+28.4%) and RMSSD (+31.5%), and the LF/HF ratio rose in both groups, but none of these shifts reached significance.

### 3.7. Sensitivity Analyses Using Continuous Adiposity Measures

When the dichotomous BMI grouping was replaced by continuous adiposity moderators, the BMI-dependent pattern was not reproduced. Baseline BMI did not moderate the change in any outcome, including fat mass (β = 0.163 kg per kg/m^2^, 95% CI −0.124 to 0.451, *p* = 0.254), percent body fat (β = 0.073, 95% CI −0.136 to 0.283, *p* = 0.479), body weight (*p* = 0.131), FMD (*p* = 0.429), aggregation index (*p* = 0.616), LDL-cholesterol (*p* = 0.894) or critical shear stress (*p* = 0.264). Baseline percent body fat likewise moderated no outcome (all *p* ≥ 0.135). The single exception was baseline fat mass, which weakly moderated the change in fat mass itself (β = 0.036 kg per kg, 95% CI 0.005 to 0.068, *p* = 0.027), an association that is partly mathematically coupled to the outcome and did not extend to percent body fat (*p* = 0.648) or body weight (*p* = 0.641). Baseline-adjusted ANCOVA on follow-up values did not alter the direction or significance of any between-group comparison. These analyses indicate that the interactions observed with the categorical WHO Asia-Pacific split are not robust to a continuous specification of adiposity, and the BMI-stratified findings should therefore be read as hypothesis-generating rather than as evidence of a graded obesity-dependent response.

## 4. Discussion

The obese group improved in isolated lipid (TC, LDL-C) and haemorheological (aggregation index, critical shear stress) markers, whereas the non-obese group showed reductions in DBP and MAP. BMD, BMC, aerobic capacity, arterial stiffness and HRV were unchanged in both groups. Effect sizes for the significant group × time interactions in body composition were medium-to-large (ηp^2^ = 0.129–0.214 by Cohen’s [32] convention), but with 14 and 18 participants per stratum these estimates are imprecise and were not corroborated when adiposity was modelled continuously (Section 3.7), so they should not be read as evidence of robust differential adaptation. Taken together, these results are compatible with within-group vascular change in both strata that cannot be attributed to mat Pilates in the absence of a control group, and they do not establish either a BMI-dependent response or a need for additional support, and they provide the first integrated physiological profile of stratified Pilates response in middle-aged women.

### 4.1. Body Composition and Aerobic Capacity

The body-composition results showed the clearest divergence between groups. Non-obese women lost 1.5% of body weight, whereas obese women gained fat mass (+2.2%) and percent body fat (+1.6%); the group × time interactions were large for fat mass (ηp^2^ = 0.214) and percent body fat (ηp^2^ = 0.177) and approached the large-effect threshold for body weight (ηp^2^ = 0.129). Because energy intake and non-exercise activity were not measured, the following mechanisms are offered only as untested hypotheses for the unexpected adiposity gain in the obese subgroup, not as established explanations. The first is energy expenditure: mat Pilates typically burns only 200–300 kcal per 60 min session at the moderate Borg 11–15 intensities used here, too little to create the negative energy balance needed for fat loss when habitual intake is preserved. ACSM evidence indicates that ≥225–420 min/week of moderate-to-vigorous aerobic exercise is required for clinically relevant weight loss (>3%) in adults with obesity [33], well above the ~180 min/week of moderate-intensity mat Pilates delivered here. A second mechanism is behavioural compensation. In the 12-week supervised exercise trial of King et al. [34] in adults with overweight or obesity, about 30% of participants showed substantially reduced weight loss attributable to compensatory eating, and Caudwell et al. [35] reported that exercise-induced hunger drive is greater in people with higher baseline adiposity. We did not measure energy intake, but the differential response; weight loss in non-obese women yet adiposity gain in obese women on the same exercise dose is consistent with greater compensatory intake in the obese subgroup. Reduced non-exercise activity thermogenesis (NEAT) is a parallel route, with metabolic-chamber studies showing 100–500 kcal/day downward compensation in some individuals during exercise programmes [36]. The third mechanism is the nature of the modality itself: mat Pilates is fundamentally a flexibility- and core-stability-oriented practice rather than a caloric-deficit one. The topical review of 14 Pilates trials in overweight or obese adults by Bertoli and colleagues [21] reported small-to-moderate reductions in adiposity, but almost always in studies that combined Pilates with dietary advice or with weight-bearing supplementary exercise. Su et al. [37] similarly reported modest body-composition change in community-dwelling middle-aged women after 12 weeks of Pilates, with the strongest gains in functional fitness rather than fat mass. Our findings fit this pattern: Pilates alone is insufficient for fat loss in obese middle-aged women but retains its vascular and metabolic benefits across the BMI spectrum.

BMD and BMC did not change in either group over 12 weeks. Mat Pilates is a low-impact, submaximal resistance modality, and the mechanical loading needed to elicit measurable bone accrual in adults usually requires a higher-impact stimulus or a longer programme [27]. The higher baseline BMD and BMC in the obese group most likely reflect the chronic weight-bearing effect of greater body mass on bone.

Aerobic capacity was unchanged in either group. This is unsurprising, since mat Pilates rarely exceeds 50–60% of HRmax and does not provide the sustained aerobic stimulus required for measurable cardiorespiratory gain [38]. The non-significant +10.4% trend in VO_2_max in the non-obese group probably reflects a small training-induced improvement in submaximal cycling efficiency rather than a true rise in maximal oxygen uptake, and should not be over-interpreted given the wide between-subject variability of the submaximal estimation procedure.

### 4.2. Cardiovascular Function

Cardiovascular outcomes diverged sharply between groups. The 15.6% rise in FMD in the non-obese group and 29.7% rise in the obese group are clinically meaningful, not merely statistically detectable. Although meta-analytic data associate a higher FMD with fewer future cardiovascular events at the population level [39,40], such epidemiological associations cannot be converted into an individual event-risk reduction produced by a 12-week uncontrolled intervention, and no such projection is made here. The mechanism most consistent with the data is sustained shear-stress stimulation of eNOS and a corresponding rise in NO bioavailability during the rhythmic breathing and low-load isometric work that characterise mat Pilates [4,15,16,17]. The obese group’s baseline FMD (7.6%) sat below the reference range reported for premenopausal women [41]; the observed absolute gain of roughly 2 percentage points is described only as a within-group change and is not interpreted as reclassifying cardiovascular-risk category. Wong et al. [42] reported a 17% rise in FMD after 12 weeks of mat Pilates in obese young women with elevated blood pressure, and our results extend that finding to middle-aged women on both sides of the BMI cut-off. The network meta-analysis of Sun and colleagues [22] ranked endurance, interval, resistance and hybrid training as effective for FMD in postmenopausal women but did not include Pilates; the present data therefore add to a thin part of the evidence base; because no aerobic comparator was studied, we do not claim equivalence between mat Pilates and traditional aerobic protocols.

Three methodological points bear on the differential FMD response. The group × time interaction did not reach significance (*p* = 0.352, ηp^2^ = 0.029), so the apparent difference should be read as a clinically suggestive pattern rather than an established effect. The lower baseline FMD in the obese group (7.6 ± 2.5% vs. 9.8 ± 2.6%; *p* = 0.021) created greater absolute room for improvement, an inverse ceiling effect that the simple time main effect cannot disentangle. Within-group variance was wide in both groups, and the sample (*n* = 32) was powered for the primary group × time effects on body composition rather than for the smaller interaction effect on FMD; on the present effect-size estimates, *n* ≥ 60 per group would be required to detect a true group × time interaction of ηp^2^ ≈ 0.03 at conventional power. Premenopausal women’s FMD is also sensitive to menstrual-cycle phase, with documented swings of 2–4 percentage points across the cycle driven by oestrogen-mediated changes in NO availability [43,44]; cycle phase was not standardised here, which would add residual variance to FMD and reduce power to detect the interaction. Despite these caveats, the within-group changes were robust (ηp^2^ = 0.458 for the time main effect, a large effect), and the clinical magnitude of the FMD gain in both groups is the more important message for exercise prescription.

DBP and MAP fell by 5.6% and 4.7% in the non-obese group, while blood pressure was unchanged in the obese group. This pattern is consistent with the modest hypotensive effect of aerobic and low-intensity resistance training in normotensive cohorts [45]; the lack of change in the obese group reflects two converging factors. Baseline blood pressure was higher in the obese group (123/75 vs. 118/72 mmHg) and was probably sustained by chronic sympathetic over-activity [3], structural arterial remodelling indexed by their higher baseline baPWV (~1290 vs. 1170 cm/s), and obesity-related sodium retention, mechanisms that low-intensity exercise alone is unlikely to reverse within 12 weeks. Dietary behaviour and habitual physical activity, which tend to shift together in adults with obesity [46], were not monitored here and may have offset any training-induced reduction in this stratum.

The unchanged baPWV in both groups is likewise expected: meta-analytic data show that arterial stiffness responds mainly to aerobic training and is largely unaffected by low-intensity resistance work over 12 weeks [47,48]. The obese group had higher baseline baPWV, in keeping with the structural remodelling associated with obesity-related cardiometabolic risk, but the low-intensity Pilates dose did not alter this profile within the trial. ABI was within the normal range and unchanged in both groups, suggesting that the central conduit-artery adaptation indexed by FMD is not paralleled by changes in peripheral arterial perfusion at this dose.

### 4.3. Blood-Metabolic Parameters

The lipid response in the obese group aligns with two recent Pilates trials in postmenopausal women with cardiometabolic risk that found TC and LDL-C improvements after 12 weeks [49], and with the broader literature on modality-dependent lipid effects in mixed populations [50,51]. The likely mechanism is the well described post-exercise enhancement of skeletal-muscle lipoprotein lipase activity and hepatic LDL-receptor up-regulation, both activated by repeated low-intensity contractile activity; the larger lipid response in the obese group most likely reflects their higher baseline TC and LDL-C and the greater biological room for improvement. The non-obese group showed no significant change in any lipid marker, consistent with their already favourable baseline profile.

Fasting glucose, insulin and HOMA-IR were unchanged in both groups. Although baseline insulin and HOMA-IR were higher in the obese group, consistent with established obesity-related insulin resistance, 12 weeks of low-intensity Pilates was insufficient to modify insulin sensitivity in either subgroup. Glucose metabolism in obese middle-aged women is typically responsive to higher-intensity aerobic or combined exercise paired with caloric restriction [33], and the modest energy demand of mat Pilates probably fell below the threshold for measurable glycaemic improvement.

### 4.4. Haemorheological Parameters

The haemorheological changes were among the more novel findings of this study. To our knowledge it is one of the first demonstrations that mat Pilates can shift erythrocyte aggregation and disaggregation thresholds in obese middle-aged women. Three mechanisms can plausibly account for the response. Repeated rhythmic diaphragmatic breathing during Pilates produces pronounced fluctuations in intrathoracic pressure and venous return, exposing red blood cells to repeated cycles of moderate shear and recovery; in exercise haemorheology this pattern enhances erythrocyte deformability through membrane lipid remodelling and Akt-pathway-dependent activation of erythrocyte NO synthase [19,20]. The lower aggregation index in the obese group is consistent with a fall in plasma fibrinogen and inflammatory cytokines (interleukin-6, tumour necrosis factor-α), which are major drivers of erythrocyte aggregation in obesity and decline with regular exercise [5,20]. The substantial reduction in critical shear stress for disaggregation (ηp^2^ = 0.376, a large time main effect) means that less mechanical force is required to break apart erythrocyte aggregates, a state that improves microvascular perfusion and tissue oxygen delivery. That this effect was strongest in obese women, whose baseline haemorheological profile is marked by elevated plasma viscosity and impaired RBC deformability [25], again points to greater biological room for improvement. EI at 3 Pa was unchanged in both groups, indicating that resting erythrocyte deformability at this shear level is less responsive to a 12-week Pilates dose than the dynamic aggregation/disaggregation properties. These changes are directly relevant to tissue oxygenation, metabolic flexibility and cardiovascular risk in obese middle-aged women, and they suggest a haemorheological change following mat Pilates that has not been described in earlier trials, although the uncontrolled design means it cannot be attributed specifically to Pilates.

### 4.5. Heart Rate Variability

HRV indices did not change significantly in either group. The descriptive shifts in the non-obese group (SDNN +28.4%, RMSSD +31.5%) are sizeable and consistent with a parasympathetic adaptation, but the wide within-group variance (RMSSD post-intervention SD 40.4 ms on a mean of 39.4 ms) reflects two sources of noise. One is the well-known day-to-day biological variability of short-term HRV recordings. The other is the absence of menstrual-cycle phase control: autonomic balance shifts substantially across the cycle in premenopausal women, with time-domain HRV indices varying by 10–20% and frequency-domain markers showing parallel fluctuations [52]. The obese group showed no such trend, possibly because their baseline pattern of higher sympathetic outflow [3] requires a stronger or longer stimulus to shift. Frequency-domain markers (LF nu, HF nu and the LF/HF ratio) likewise showed no significant change, although the LF/HF ratio rose numerically in both groups (NOG +18.9%, OG +25.1%), a pattern that could reflect either a small sympathetic shift or measurement noise in the small sample. Earlier reports of HRV gain after Pilates have been mixed and tend to favour programmes of higher volume or longer duration [14,53].

### 4.6. Practical Implications

Mat Pilates is accessible, needs minimal equipment and is well tolerated in middle-aged women; no adverse events occurred in our cohort and attendance averaged above 94%. The data support three recommendations. In non-obese middle-aged women with elevated cardiovascular risk markers (mildly raised blood pressure, suboptimal FMD), mat Pilates may be a useful low-impact option and was associated with improved FMD and resting blood pressure; however, because the study lacked a control group, it cannot be established as a stand-alone vascular-protective treatment, and this should be confirmed in controlled trials. In obese middle-aged women, the divergent body-composition response suggests—although the present study did not test this—that combining mat Pilates with additional strategies may be needed, for example: a moderate hypocaloric diet (about −300 to −500 kcal/day from habitual intake) and a supplementary aerobic component (≥150 min/week of moderate-intensity aerobic activity in addition to Pilates), in line with current ACSM guidance for weight management [33,38]. The differential body-composition response in our trial suggests that without such supplementation, obese women may experience adiposity drift despite good adherence. Finally, the haemorheological changes in obese women are hypothesis-generating for future study of mat Pilates in women with microvascular or peripheral circulatory complaints; a direct clinical benefit for tissue perfusion was not measured here and should not be assumed. Across all of these uses, the stratified design points to a broader principle for exercise prescription in midlife: identical protocols can yield systematically different physiological returns depending on baseline cardiometabolic status, and outcome expectations should be calibrated accordingly.

### 4.7. Strengths and Limitations

The principal strength of this study is the breadth of physiological profiling: DXA body composition, BMD/BMC, FMD, baPWV, fasting lipid and glucose profiles, haemorheological function and time- and frequency-domain HRV were collected on the same participants under a single supervised programme. The stratified design allowed a direct contrast between non-obese and obese women receiving an identical intervention, which is rare in the Pilates literature. Adherence was high (94.2%), every session was supervised by one certified instructor with more than 10 years of experience, which improved protocol fidelity, and outcome assessors were blinded to time point. To our knowledge this is also the first study to report differential haemorheological adaptation to mat Pilates in middle-aged women across the BMI spectrum.

Hormonal fluctuations are known to shift FMD by 2–4 percentage points and time-domain HRV by 10–20% across the cycle in premenopausal women [43,44,52]; this probably contributed to the wide variance in FMD and HRV and to the FMD group × time interaction failing to reach significance. The analytical sample (*n* = 32) was adequate for the primary body-composition effects but underpowered for the smaller effects on HRV and the FMD interaction; on present estimates, approximately 60 participants per group would be required to detect a group × time FMD interaction of ηp^2^ ≈ 0.03 at conventional power. HRV frequency-domain values are reported in normalised units rather than absolute ms^2^ power, which limits cross-study comparison. Seasonal effects on vascular function (FMD typically rises in summer) were not controlled, although the parallel timing of pre- and post-intervention measurements in both groups should have minimised systematic bias. Several potential confounders were not objectively monitored. Dietary intake was not recorded with weighed food diaries or 24 h recalls, habitual non-exercise physical activity was not measured by accelerometry, sleep duration and quality were not assessed, and menstrual-cycle phase was neither standardised nor recorded at the time of testing. This matters beyond energy balance alone, because diet quality and physical activity have been shown to track with coordinated shifts in blood-metabolic pathway activity [18], so unmeasured dietary variation could contribute to the metabolic differences observed here. Each of these can independently influence the outcomes reported: energy balance and non-exercise activity thermogenesis act directly on body composition; sleep restriction alters insulin sensitivity, lipid handling and cardiac autonomic balance; and cycle phase modulates FMD, blood pressure and heart rate variability in premenopausal women. The body-composition, metabolic, HRV and vascular findings must therefore be interpreted with these unmeasured influences in mind. In addition, the dichotomous WHO Asia-Pacific BMI cut-off is a crude proxy for adiposity that cannot distinguish fat distribution or fat-free mass and may mask heterogeneity within strata; the continuous-moderator sensitivity analyses reported in Section 3.7 did not reproduce the categorical findings, reinforcing this caution. Waist circumference and waist-to-height ratio were not available in the original dataset and could not be examined. Finally, the single instructor improved internal consistency but limits generalisability to community settings with multiple instructors of varying experience. Because the design was uncontrolled and single-arm, part of the observed within-group change—particularly in participants with extreme baseline values—may reflect regression to the mean, which cannot be excluded without a non-exercising control group; the baseline-adjusted ANCOVA reported above partially addresses baseline imbalance but does not remove this threat. Future work should include a prospective trial with a non-exercising control arm and a priori BMI stratification, objective dietary monitoring, hormonal profiling with menstrual-phase standardisation, longer intervention (24–48 weeks) to test maintenance of vascular gains, direct comparison of Pilates with HIIT and progressive resistance training, and mechanistic biomarker panels (NO metabolites, eNOS expression, inflammatory cytokines, plasma fibrinogen) to test the proposed pathways directly.

## 5. Conclusions

A 12-week supervised mat Pilates programme was associated with improved brachial-artery FMD in both non-obese and obese middle-aged women, although the absence of a control group precludes causal inference; the group × time interaction for FMD was not significant, so a larger gain in the obese group is only suggested, not established. The groups diverged elsewhere: non-obese women lost a small amount of body weight and showed reductions in DBP and MAP, while obese women gained slightly in fat mass and percent body fat but improved their lipid profile, aggregation index and critical shear stress. Bone status, aerobic capacity, arterial stiffness and HRV were unchanged in both groups. Because none of these between-group contrasts survived formal interaction testing, and because the apparent BMI dependence was not reproduced when adiposity was modelled continuously, these observations should be regarded as hypothesis-generating only. Mat Pilates may therefore be a feasible and well tolerated option for middle-aged women, but the present data cannot establish it as an effective vascular- or lipid-targeted modality, nor demonstrate that its effects differ by obesity status; obese middle-aged women may still require adjunct strategies, such as dietary modification, higher-intensity aerobic loading or longer training, if broader cardiometabolic and autonomic gains are required. Adequately powered randomised controlled trials with a non-exercising comparator, a priori BMI or adiposity stratification, and objective monitoring of diet, non-exercise physical activity, sleep and menstrual-cycle phase are required before any BMI-dependent adaptation to mat Pilates can be considered established.

## Figures and Tables

**Table 1:** Participant characteristics at baseline.

Variable	NOG (*n* = 14)	OG (*n* = 18)	*p*
Age (years)	46.4 ± 8.0	48.4 ± 7.4	0.464
Height (cm)	159.2 ± 6.0	158.4 ± 5.7	0.721
Body weight (kg)	58.5 ± 4.3	70.6 ± 9.4	<0.001 ***
BMI (kg/m^2^)	22.9 ± 1.1	28.0 ± 3.3	<0.001 ***
Fat-free mass (kg)	36.3 ± 3.4	39.1 ± 4.7	0.058
Percent body fat (%)	34.6 ± 3.2	41.3 ± 5.0	<0.001 ***

Values are mean ± SD. Between-group comparisons used independent *t*-tests. *** *p* < 0.001. BMI, body mass index; NOG, non-obese group; OG, obese group; SD, standard deviation.

**Table 2:** DXA-measured body composition and aerobic exercise capacity before and after the 12-week mat Pilates intervention.

Variable	NOG (*n* = 14)			OG (*n* = 18)			*p* (ηp^2^)		
	Pre	Post	Cohen’s d [95% CI]	Pre	Post	Cohen’s d [95% CI]	Group	Time	G × T
*DXA-measured body composition*									
Body weight (kg)	58.5 ± 4.3	57.6 ± 4.1	−0.21 * [−0.41, −0.03]	70.6 ± 9.4	71.0 ± 10.1	+0.04 [−0.05, 0.11]	<0.001 (0.412)	0.541 (0.012)	0.044 (0.129)
BMI (kg/m^2^)	22.9 ± 1.1	22.6 ± 1.2	−0.27 [−0.58, 0.01]	28.0 ± 3.3	28.0 ± 3.3	+0.01 [−0.07, 0.11]	<0.001 (0.519)	0.266 (0.041)	0.096 (0.089)
FFM (kg)	36.3 ± 3.4	36.0 ± 3.6	−0.10 [−0.27, 0.05]	39.1 ± 4.7	38.8 ± 4.8	−0.07 [−0.21, 0.05]	0.075 (0.102)	0.105 (0.082)	0.971 (0.000)
Fat mass (kg)	20.2 ± 2.1	19.7 ± 2.4	−0.23 [−0.64, 0.05]	29.4 ± 6.7	30.0 ± 7.0	+0.09 * [0.02, 0.23]	<0.001 (0.467)	0.556 (0.011)	0.008 (0.214)
Body fat (%)	34.6 ± 3.2	34.2 ± 3.8	−0.13 [−0.40, 0.08]	41.3 ± 5.0	42.0 ± 4.9	+0.14 * [0.03, 0.34]	<0.001 (0.421)	0.478 (0.016)	0.017 (0.177)
BMD (g/cm^2^)	1.087 ± 0.103	1.093 ± 0.106	+0.06 [−0.00, 0.15]	1.182 ± 0.110	1.181 ± 0.108	−0.01 [−0.10, 0.06]	0.023 (0.161)	0.397 (0.023)	0.209 (0.052)
BMC (g)	1966.9 ± 197.0	1964.3 ± 193.7	−0.01 [−0.08, 0.05]	2144.2 ± 252.3	2158.4 ± 270.8	+0.05 [−0.02, 0.17]	0.034 (0.141)	0.346 (0.029)	0.252 (0.044)
*Aerobic exercise capacity*									
VO_2_max (mL/kg/min)	25.6 ± 3.8	28.3 ± 6.8	+0.50 [0.05, 1.00]	23.6 ± 3.7	23.8 ± 4.4	+0.03 [−0.38, 0.51]	0.142 (0.071)	0.153 (0.065)	0.144 (0.070)
PWC75% (W)	73.1 ± 24.0	78.0 ± 24.7	+0.20 [−0.08, 0.66]	84.2 ± 19.2	86.8 ± 22.4	+0.12 [−0.10, 0.46]	0.154 (0.066)	0.108 (0.081)	0.604 (0.009)

Values are mean ± SD; effect sizes (ηp^2^) in parentheses. * *p* < 0.05 within-group significant change. BMC, bone mineral content; BMD, bone mineral density; DXA, dual-energy X-ray absorptiometry; FFM, fat-free mass; G, group; G × T, group × time interaction; NOG, non-obese group; OG, obese group; PWC75%, physical work capacity at 75% of maximal heart rate; T, time; VO_2_max, maximal oxygen uptake.

**Table 3:** Cardiovascular function before and after the 12-week mat Pilates intervention.

Variable	NOG (*n* = 14)			OG (*n* = 18)			*p* (ηp^2^)		
	Pre	Post	Cohen’s d [95% CI]	Pre	Post	Cohen’s d [95% CI]	Group	Time	G × T
*Resting blood pressure*									
SBP (mmHg)	118.0 ± 12.3	113.6 ± 11.8	−0.36 [−0.90, 0.04]	121.7 ± 13.9	122.6 ± 11.2	+0.07 [−0.41, 0.48]	0.434 (0.021)	0.474 (0.017)	0.183 (0.058)
DBP (mmHg)	71.8 ± 6.9	67.8 ± 6.6	−0.60 * [−1.17, −0.07]	74.6 ± 11.3	72.5 ± 8.6	−0.21 [−0.68, 0.17]	0.432 (0.021)	0.046 (0.123)	0.501 (0.015)
MAP (mmHg)	87.2 ± 8.1	83.1 ± 6.9	−0.55 [−1.21, −0.06]	90.3 ± 11.5	89.2 ± 8.8	−0.11 [−0.60, 0.30]	0.405 (0.023)	0.116 (0.078)	0.322 (0.033)
PP (mmHg)	46.2 ± 8.6	45.9 ± 11.2	−0.03 [−0.45, 0.32]	47.2 ± 8.4	50.1 ± 7.6	+0.37 [−0.01, 0.87]	0.742 (0.004)	0.207 (0.051)	0.177 (0.060)
*Arterial stiffness*									
baPWV-R (cm/s)	1171.8 ± 97.4	1168.3 ± 88.0	−0.04 [−0.32, 0.23]	1302.4 ± 171.5	1282.1 ± 184.6	−0.11 [−0.51, 0.17]	0.016 (0.177)	0.420 (0.021)	0.607 (0.009)
baPWV-L (cm/s)	1173.3 ± 108.7	1160.0 ± 96.5	−0.13 [−0.52, 0.20]	1282.1 ± 169.7	1252.3 ± 203.8	−0.16 [−0.61, 0.16]	0.046 (0.127)	0.277 (0.038)	0.694 (0.005)
ABI-R	1.16 ± 0.07	1.21 ± 0.07	+0.80 [−0.02, 2.09]	1.19 ± 0.09	1.20 ± 0.09	+0.13 [−0.28, 0.55]	0.343 (0.030)	0.059 (0.110)	0.192 (0.056)
ABI-L	1.15 ± 0.06	1.18 ± 0.11	+0.35 [−0.46, 1.87]	1.15 ± 0.10	1.19 ± 0.08	+0.38 [0.10, 0.80]	0.920 (0.000)	0.092 (0.089)	0.924 (0.000)
*Endothelial function*									
FMD (%)	9.8 ± 2.6	11.3 ± 3.6	+0.49 * [0.22, 0.97]	7.6 ± 2.5	9.8 ± 2.3	+0.93 ** [0.48, 1.88]	0.021 (0.164)	<0.001 (0.458)	0.352 (0.029)
Baseline diameter (mm)	3.45 ± 0.30	3.47 ± 0.31	+0.07 [−0.15, 0.31]	3.57 ± 0.43	3.62 ± 0.38	+0.11 [−0.09, 0.28]	0.371 (0.027)	0.204 (0.052)	0.644 (0.007)

Values are mean ± SD; effect sizes (ηp^2^) in parentheses. * *p* < 0.05; ** *p* < 0.01 within-group significant change. ABI, ankle–brachial index; baPWV, brachial–ankle pulse wave velocity; DBP, diastolic blood pressure; FMD, flow-mediated dilation; G, group; G × T, group × time interaction; L, left; MAP, mean arterial pressure; NOG, non-obese group; OG, obese group; PP, pulse pressure; R, right; SBP, systolic blood pressure; T, time.

**Table 4:** Blood-metabolic parameters before and after the 12-week mat Pilates intervention.

Variable	NOG (*n* = 14)			OG (*n* = 18)			*p* (ηp^2^)		
	Pre	Post	Cohen’s d [95% CI]	Pre	Post	Cohen’s d [95% CI]	Group	Time	G × T
TC (mg/dL)	195.6 ± 49.6	189.8 ± 30.7	−0.15 [−0.52, 0.36]	212.2 ± 39.6	199.4 ± 33.7	−0.35 * [−0.93, −0.10]	0.302 (0.035)	0.038 (0.132)	0.453 (0.019)
TG (mg/dL)	104.6 ± 34.9	99.7 ± 30.8	−0.15 [−0.79, 0.50]	123.8 ± 36.2	115.9 ± 40.1	−0.20 [−0.68, 0.12]	0.143 (0.070)	0.255 (0.042)	0.805 (0.002)
HDL-C (mg/dL)	60.8 ± 12.2	60.8 ± 11.9	+0.00 [−0.40, 0.37]	56.2 ± 11.1	55.0 ± 13.1	−0.10 [−0.40, 0.16]	0.274 (0.040)	0.604 (0.009)	0.652 (0.007)
LDL-C (mg/dL)	118.7 ± 47.6	115.6 ± 52.4	−0.06 [−0.57, 0.18]	139.2 ± 32.2	126.6 ± 35.1	−0.37 * [−0.82, −0.11]	0.158 (0.065)	0.047 (0.121)	0.253 (0.043)
Glucose (mg/dL)	92.5 ± 13.3	93.7 ± 15.6	+0.08 [−0.54, 0.58]	100.0 ± 19.4	101.3 ± 14.7	+0.07 [−0.22, 0.46]	0.223 (0.049)	0.572 (0.010)	0.991 (0.000)
Insulin (μU/mL)	5.4 ± 2.6	5.2 ± 2.2	−0.05 [−0.48, 0.36]	7.7 ± 3.0	8.4 ± 3.5	+0.21 [−0.15, 0.60]	0.023 (0.161)	0.410 (0.022)	0.325 (0.032)
HOMA-IR	1.25 ± 0.70	1.26 ± 0.75	+0.01 [−0.49, 0.30]	1.99 ± 1.00	2.16 ± 1.06	+0.16 [−0.17, 0.51]	0.026 (0.154)	0.392 (0.024)	0.472 (0.017)

Values are mean ± SD; effect sizes (ηp^2^) in parentheses. * *p* < 0.05 within-group significant change. G, group; G × T, group × time interaction; HDL-C, high-density lipoprotein cholesterol; HOMA-IR, homeostasis model assessment of insulin resistance; LDL-C, low-density lipoprotein cholesterol; NOG, non-obese group; OG, obese group; T, time; TC, total cholesterol; TG, triglycerides.

**Table 5:** Haemorheological parameters before and after the 12-week mat Pilates intervention.

Variable	NOG (*n* = 14)			OG (*n* = 18)			*p* (ηp^2^)		
	Pre	Post	Cohen’s d [95% CI]	Pre	Post	Cohen’s d [95% CI]	Group	Time	G × T
Aggregation index (%)	38.7 ± 6.8	38.7 ± 6.5	−0.00 [−0.40, 0.37]	42.3 ± 7.0	39.1 ± 5.6	−0.52 * [−0.97, −0.20]	0.156 (0.066)	0.036 (0.135)	0.056 (0.117)
RBC deformability—EI at 3 Pa	0.34 ± 0.01	0.33 ± 0.02	−0.24 [−0.90, 0.48]	0.33 ± 0.01	0.33 ± 0.02	+0.04 [−0.53, 0.64]	0.262 (0.042)	0.725 (0.004)	0.530 (0.013)
Critical shear stress (mPa)	427.7 ± 178.6	338.1 ± 115.4	−0.61 [−1.34, −0.02]	518.6 ± 167.2	386.1 ± 129.8	−0.89 * [−1.59, −0.49]	0.149 (0.068)	<0.001 (0.376)	0.429 (0.021)

Values are mean ± SD; effect sizes (ηp^2^) in parentheses. * *p* < 0.05 within-group significant change. EI, elongation index; G, group; G × T, group × time interaction; NOG, non-obese group; OG, obese group; RBC, red blood cell; T, time.

**Table 6:** Heart rate variability parameters before and after the 12-week mat Pilates intervention.

Variable	NOG (*n* = 14)			OG (*n* = 18)			*p* (ηp^2^)		
	Pre	Post	Cohen’s d [95% CI]	Pre	Post	Cohen’s d [95% CI]	Group	Time	G × T
*Time-domain measures*									
Mean RR (ms)	929.8 ± 99.9	951.8 ± 150.9	+0.17 [−0.42, 1.02]	923.6 ± 118.9	934.8 ± 116.2	+0.09 [−0.41, 0.53]	0.753 (0.003)	0.498 (0.015)	0.821 (0.002)
SDNN (ms)	25.8 ± 12.8	33.2 ± 25.1	+0.39 [−0.10, 0.85]	24.7 ± 15.7	22.2 ± 6.4	−0.23 [−0.70, 0.41]	0.218 (0.050)	0.561 (0.011)	0.115 (0.081)
RMSSD (ms)	29.9 ± 16.7	39.4 ± 40.4	+0.33 [−0.38, 0.82]	32.9 ± 26.0	26.2 ± 9.2	−0.38 [−0.79, 0.16]	0.491 (0.016)	0.939 (0.000)	0.124 (0.077)
*Frequency-domain measures*									
LF (nu)	43.9 ± 20.4	50.9 ± 20.6	+0.34 [−0.20, 1.06]	37.5 ± 19.6	38.4 ± 21.8	+0.04 [−0.51, 0.60]	0.131 (0.075)	0.382 (0.025)	0.464 (0.018)
HF (nu)	56.1 ± 20.4	49.1 ± 20.6	−0.34 [−1.03, 0.18]	62.5 ± 19.6	61.6 ± 21.8	−0.04 [−0.59, 0.50]	0.131 (0.075)	0.382 (0.025)	0.464 (0.018)
LF/HF ratio	1.174 ± 1.387	1.395 ± 0.955	+0.19 [−0.31, 1.19]	0.813 ± 0.775	1.017 ± 1.453	+0.18 [−0.58, 0.75]	0.264 (0.041)	0.419 (0.021)	0.974 (0.000)

Values are mean ± SD; effect sizes (ηp^2^) in parentheses. G, group; G × T, group × time interaction; HF, high-frequency power; LF, low-frequency power; NOG, non-obese group; nu, normalised units; OG, obese group; RMSSD, root mean square of successive RR differences; SDNN, standard deviation of NN intervals; T, time.

## Data Availability

De-identified participant-level data, a data dictionary and the analysis code are available from the corresponding author upon reasonable request, subject to the approval of the Institutional Review Board of Dongseo University and to applicable data-protection requirements.

## Abbreviations

The following abbreviations are used in this manuscript:

ABI	ankle–brachial index
AI	aggregation index
baPWV	brachial–ankle pulse wave velocity
BMC	bone mineral content
BMD	bone mineral density
BMI	body mass index
DBP	diastolic blood pressure
DXA	dual-energy X-ray absorptiometry
EI	elongation index
eNOS	endothelial nitric oxide synthase
FFM	fat-free mass
FMD	flow-mediated dilation
HDL-C	high-density lipoprotein cholesterol
HOMA-IR	homeostasis model assessment of insulin resistance
HRV	heart rate variability
LDL-C	low-density lipoprotein cholesterol
MAP	mean arterial pressure
NO	nitric oxide
NOG	non-obese group
OG	obese group
PP	pulse pressure
PWC75%	physical work capacity at 75% of maximal heart rate
RPE	rating of perceived exertion
SBP	systolic blood pressure
SDNN	standard deviation of NN intervals
RMSSD	root mean square of successive RR differences
TC	total cholesterol
TG	triglycerides
VO_2_max	maximal oxygen uptake
